# Improving China’s maritime law enforcement operations against overfishing in the South China Sea—Based on a comparison with the Indonesian law enforcement system against IUU Fishing

**DOI:** 10.1371/journal.pone.0319525

**Published:** 2025-04-15

**Authors:** Minyou Yu, Weizhe Liu

**Affiliations:** Wuhan University China Institute of Boundary and Ocean Studies, Wuhan University, Wuhan, China; National Cheng Kung University, TAIWAN

## Abstract

Overfishing in the South China Sea has become increasingly severe these years, with Illegal, Unreported, and Unregulated (IUU) fishing being one of the main contributors to this problem. To ensure regional and global food security as well as stability, it is necessary and urgent for the South China Sea coastal states to deal with overfishing effectively. Based on a comparison with the Indonesian law enforcement system against IUU fishing, the paper proposes a path for China to improve its fishery law enforcement system. It also utilizes VIIRS-DNB data analysis to support some viewpoints and highlight areas needing special attention. In the future, China should clarify the legislation, policy, and institutional authority distribution for fishery law enforcement; adopt more effective measures while meeting the necessity requirements of the United Nations Convention on the Law of the Sea; strengthen international cooperation, attempting to reach temporary fishing zone agreements with other coastal states in disputed waters; and involve local fishing communities in the development of the enforcement system.

## Introduction

The South China Sea (SCS) is the largest marginal sea in Southeast Asia, covering an area of about 3.5 million square kilometers. It is bordered to the south by the Sulu Sea and the Java Sea, connects to the East China Sea to the north via the Taiwan Strait, and links to the Pacific Ocean through the Luzon Strait [1]. The South China Sea is home to a vast variety of fishery resources with extremely high species diversity, making it one of the top five fishing areas in the world, with catches accounting for about 12% of the global total [2]. Overfishing in the SCS has become increasingly prominent in recent years, with Illegal, Unreported, and Unregulated (IUU) fishing being one of the main causes. Besides threatening the sustainability of maritime fishery resources, IUU fishing also poses risks to maritime security and is likely to trigger conflicts in surrounding areas [3–5]. In addition, overlapping EEZ claims in the SCS cause a series of fishery disputes [6]. If IUU fishing and fishery disputes were not properly managed, overfishing would not be remitted, and tensions could escalate in the SCS.

Chinese government adopts the term ‘illegal fishing’, which has a narrower scope, instead of IUU fishing. As defined by China, according to international law and practices combined with its national conditions, illegal fishing refers to fishing activities that violate domestic laws and regulations, typically applicable to waters under Chinese jurisdiction. IUU fishing is defined by its ‘illegal’ nature and is primarily manifested in three forms: Fishing activities are conducted within a state’s jurisdictional waters without adhering to laws, regulations, or permission of the coastal state; Fishing activities fail to comply with RFMO’s rules in the high seas when the flag state of the vessel is a certain RFMO; Fishing activities violate the obligations to conserve marine living resources under general international law in unregulated waters where no specific fishery management measures are in place. There is a debate within the academic community about whether there are high seas in SCS. Some hold that due to the geographical characteristics and extensive overlap of EEZs of the coastal states, there are no high seas in the SCS [7]. Others support the notion that parts of the SCS align with the definition of high seas [8]. This view argues that even with overlapping EEZs, some areas may not fall under any state’s EEZ. This article adopts the mainstream academic perspective, asserting that most of the SCS is covered by the EEZs of the coastal states; hence, there are no high seas. Therefore, the discussion focuses on IUU fishing within a state’s EEZ. This scope overlaps with the scope of illegal fishing as defined by China; therefore, the concepts of IUU fishing and illegal fishing in this paper are interchangeable. Regarding overlapping EEZ claims in the SCS, fishery disputes within disputed waters also need special attention. The paper intends to propose a Chinese solution to address overfishing through law enforcement operations in the SCS. Based on Chinese official stance and practices, a comprehensive law enforcement system against overfishing should contain two vital parts dealing with illegal fishing and fishery disputes.

Research related to fishery law enforcement largely falls within the social science field. For instance, Shi holds the opinion that coastal states in the SCS could develop a “gradual” new model for fishery law enforcement cooperation [9]; Shen and Huang suggest that In addition to strengthening domestic legal framework of combating IUU fishing, it is also essential to consider international cooperation and to focus on training and educating fishermen [10]. Hsiao suggests that a jointly-managed Provisional Measures Zone on maritime law enforcement and fishery issues should be established in SCS [11]; Young thinks that import prohibitions and other trade measures are often unilaterally enacted, but their effectiveness could be enhanced through collective or regional arrangements [12]. Few of these studies focus specifically on law enforcement.

Some natural science researches reflect trends of fishing intensity, most of which use two types of data—VIIRS-DNB data and AIS/VMS data provided by the Global Fishing Watch platform. These two kinds of data show high consistency on monthly and annual scales [13]. For example, based on VIIRS data, fishing trends during the spring season from 2012 to 2020 in the open-SCS fishing zone can be determined, and specific distribution patterns of certain types of fishing vessels in the open-SCS can be obtained through algorithms [14]. VMS data can reflect fishing intensity in different regions and offer solutions for quantifying and managing ecosystem disturbances [15]. Oozeki has attempted to combine VIIRS-DNB data with AIS data to reflect the intensity of IUU fishing, but the extensive application of this method faces particular challenges [16]. Due to the “unreported” nature of IUU fishing, obtaining accurate data on IUU fishing vessels is difficult, making it nearly impossible to monitor the intensity of IUU fishing directly. A common practice is to use VIIRS-DNB or VMS/AIS data to reflect fishing intensity, which is considered to have the potential for monitoring and combating IUU fishing—the European Union has been monitoring IUU fishing by the fishing intensity reflected in AIS data [17]. This paper follows the logic, using overall fishing intensity as an indicator of IUU fishing. Although data analysis cannot directly reflect the intensity of IUU fishing, it can be an indication.

On November 9, 2024, China and Indonesia issued a joint statement on advancing their comprehensive strategic partnership and building a China-Indonesia community of shared future. The two sides emphasized that maritime cooperation is a key component of their comprehensive strategic cooperation. The statement also specifically highlighted their commitment to actively promoting the institutionalized cooperation between their coast guards and deepening collaboration in law enforcement capacity building. Comparing the two states’ enforcement systems in combating illegal fishing will help them achieve these goals based on mutual understanding. Moreover, Indonesia is a pioneer in the fight against IUU fishing, which is highlighted in reports by World Bank and FAO [18,19]. Additionally, data analysis in the paper shows Indonesia’s notable success in combating IUU fishing. Given these factors, it is believed that Indonesia’s experiences can serve as a valuable reference for China, therefore, Indonesia is chosen as a comparison in the paper. The paper aims to compare the fishery law enforcement system in China and that of Indonesia against IUU fishing by conducting a normative legal analysis. It evaluates the strengths and weaknesses of each system and proposes a path for China to improve its fishery law enforcement system against overfishing in the SCS from the perspective of International Law. It also utilizes fishing intensity based on VIIRS-DNB data to support certain viewpoints. Lastly, it concludes that China should improve its fishery law enforcement system in SCS by adopting clear legislation, policies and institutional arrangements, taking more effective measures, reinforcing international cooperation and enhancing participation of local fishing communities.

## Study area and data sources

### Study area

The study area covers the nine-dash line claimed by China and the EEZ claimed by Indonesia. The sovereignty rights within the nine-dash line and their interpretation have sparked extensive debate in international law academia, by which fishery resource management and maritime law enforcement within the scope are affected in reality. Also, the nine-dash line is closely related to some fishery disputes because of the overlapping claims around it. For these reasons, the paper chose the area within the Nine-Dash Line as one of its study areas. Data analysis regarding the Indonesian EEZ aims to provide more apparent reflections on the effectiveness of its law enforcement measures. The overlapping maritime area around the Natuna Islands, which has witnessed several standoffs, has also been paid particular attention to.

### Data sources

Google Earth Engine provides the VIIRS-DNB data used in the paper. This platform offers a web-based code editor for writing and running programs, along with a robust spatial analysis library that allows users to conduct geographical spatial analysis [20]. Global Fishing Watch (GFW) is a dataset based on Automatic Identification System (AIS) records of significant global fishing activities. Studies indicate a high level of consistency between GFW data and VIIRS-DNB data [21]. In other words, VIIRS-DNB data shows a high level of consistency with global fishing intensity. Therefore, selecting VIIRS-DNB data as the data source to reflect fishing intensity is justified. The paper utilizes the Google Earth Engine platform to extract monthly VIIRS-DNB average radiance (avg_rad) data from 2012 to 2023 and analyzes trends in fishing intensity. The results are cropped to emphasize different areas.

## Research methods

### Normative legal research

The paper adopts a normative legal research method. First, it collects laws, policies, and research articles. Second, it elucidates the background and current situation of the problem to be addressed. Third, it presents the strengths and weaknesses of the enforcement systems of the two countries through comparative analysis. Finally, it synthesizes the collected materials and the data analysis results to propose possible paths for improvement in China.

### Mann-Kendall (M-K) test

The Mann-Kendall (M-K) test is commonly used to detect significant trends in time series data [22]. As a result, it is widely employed in examining trends in variables such as precipitation, runoff, temperature, water quality, and more. In this paper, M-K test is used to examine the trends in fishing intensity. Trends are sorted into five categories, namely, highly significant decrease, significant decrease, non-significant variation, significant increase and highly significant increase.

### Interdisciplinary research

Science is both highly differentiated and integrated. Issues like overfishing in SCS, which may impact regional and even global food security, inevitably involve knowledge and methods from multiple disciplines. The paper proposes suggestions for improving Chinese law enforcement system against overfishing in the SCS from a perspective that combines geographical information science and international law. It adopts analytical methods from natural science and international law, aiming to leverage the strengths of both disciplines to form practical and feasible recommendations.

## Results

**Fig 1:**
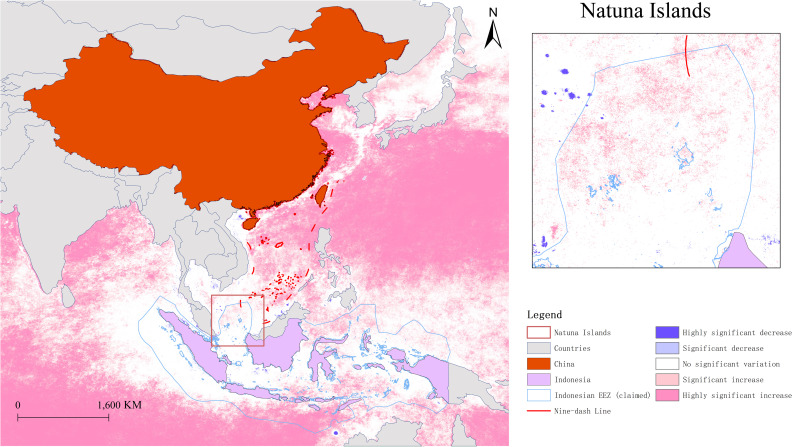
Fishing intensity trend from 2019–2023 in the South China Sea. (Source of the data: https://eogdata.mines.edu/nighttime_light/monthly/v10/2019/ to https://eogdata.mines.edu/nighttime_light/monthly/v10/2023/).

The Mann-Kendall test reflects the trends and spatial distribution of fishing intensity in the study area from 2019 to 2023 (Fig 1).

It is evident from Fig 1 that areas with increased fishing intensity far outnumber those with decreased intensity. Significantly decreased areas are sparsely distributed throughout the study area without a clear pattern. Most of the pixels within the nine-dash line show a highly significant increase trend. It is estimated that since the 1950s, the total amount of fishery resources in the SCS has declined by 70%–95% [23].

**Fig 2:**
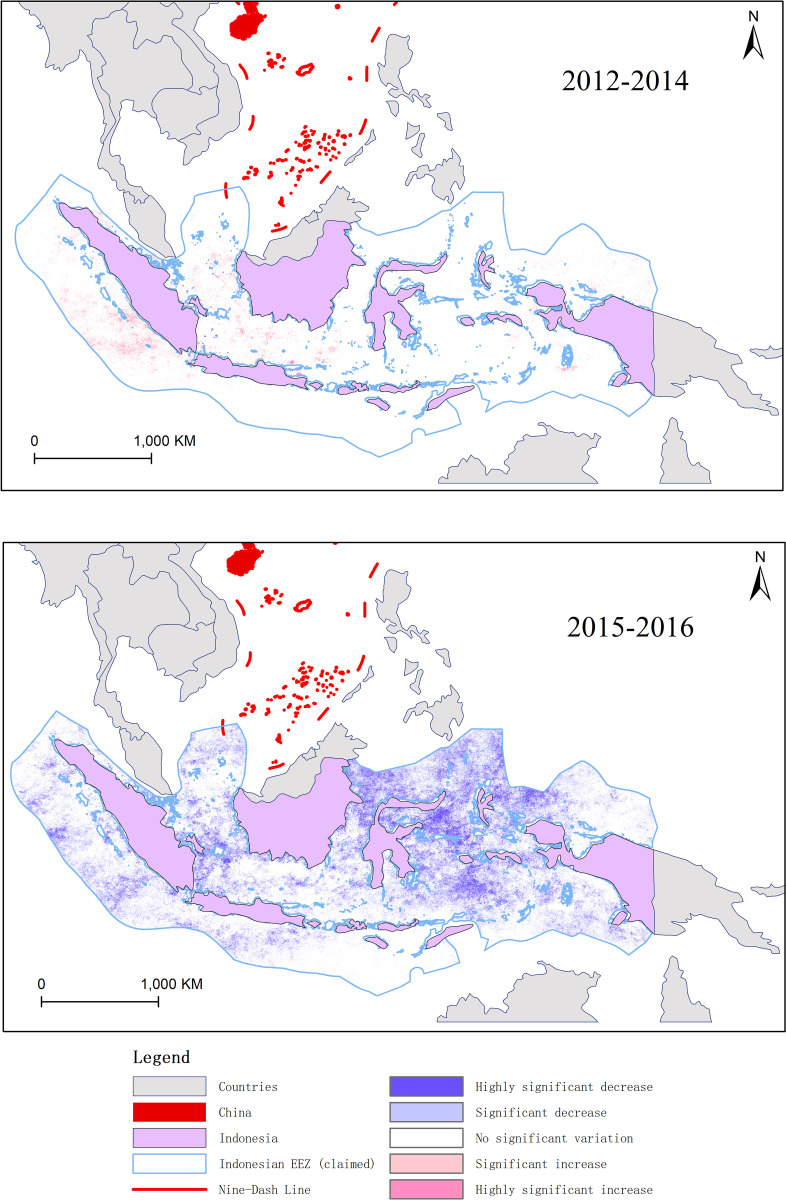
Contrast of fishing intensity within Indonesian EEZ. (Source of the data: https://eogdata.mines.edu/nighttime_light/monthly/v10/2012/ to https://eogdata.mines.edu/nighttime_light/monthly/v10/2016/).

The data analysis also illustrates the effectiveness of Indonesian measures to combat IUU fishing. In October 2014, Susi Pudjiastuti, the former Minister of Indonesian Ministry of Maritime Affairs and Fisheries (KKP), took a series of measures to crack down on IUU fishing in Indonesian waters. Prior to Susi’s tenure, from 2012 to October 2014, there was no significant change in overall fishing intensity within the Indonesian EEZ, with a few areas showing a noticeable increasing trend (Fig 2). However, after Susi took office and implemented strict measures, from 2015 to 2016, there was a significant decrease in fishing intensity within the Indonesian EEZ.

**Fig 3:**
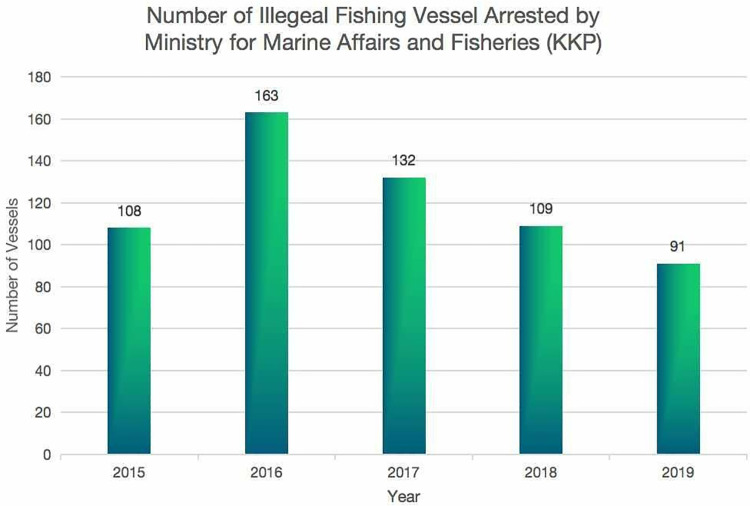
Number of illegal fishing vessels arrested by KKP.

The statistical data indicates that the number of vessels arrested by KKP peaked in 2016. From then till 2019, the number of arrested vessels decreased annually (Fig 3), which may also be attributed to Susi’s stringent measures.

To avoid the depletion of fishery resources, it is urgent and necessary for SCS coastal states to handle IUU fishing and fishing disputes properly. Since the analysis of fishing intensity and statistical data reflects the significant proportion of IUU fishing in Indonesian overall fishing activities and demonstrates the effectiveness of Indonesian law enforcement measures, Indonesia offers precious research value.

## Discussion

### Comparison of fishery law enforcement systems between Indonesia and China

Being the largest archipelago country in the world, Indonesia is recognized as a pioneer in the global fight against IUU fishing. In May 2014, during his television election campaign, Indonesian President Joko Widodo introduced the “Global Maritime Fulcrum” strategy. In October 2014, upon assuming the presidency, he further elaborated on the essence of the strategy, which includes combating IUU fishing to protect marine resources and national interests. The 2015 Indonesian Defense White Paper identified IUU fishing as a national threat. Given the archipelagic nature, its dependence on fishery, and the need to secure its fishing rights, combating IUU fishing holds strategic significance for Indonesia. Indonesia has taken substantial measures to build its law enforcement system, which has been proven effective. Comparing its system with China’s may provide insights that could benefit China. As Confucius said, ‘Adopt the good and reform the bad’.

#### Legal framework and institutional arrangement.

The Indonesian legal system evolved from the old legal system of the Dutch East Indies colonial government, where most colonial laws have been replaced by new ones, while some old ones are still in use [24]. The Indonesian legal system is extremely complex, with legislation (excluding ministerial regulations) divided into seven levels. Since gaining independence in 1945, Indonesia has enacted over 1600 laws, making its legal system one of the “largest legal systems in the world.”

The complexity is also reflected in maritime security, where there are 17 laws related to the subject, including the Maritime Act, Ocean Affairs Act, Indonesian Waters Act, and Shipping Act. The existing legal framework covers most maritime security affairs, but due to a lack of coordination, there are conflicts between laws. For example, Article 24 of the Indonesian Waters Act stipulates that various agencies, including the Navy, Coast Guard, Ministry of Transportation, Customs, and Immigration, conduct enforcement activities in all Indonesian waters. In contrast, Articles 59 to 62 of the Maritime Act state that the safety, supervision, and enforcement activities in Indonesian jurisdictional waters are the responsibilities of the Maritime Security Agency (BAKAMLA). Such conflicts lead to inefficient maritime law enforcement, as different agencies enforce laws based on different statutes without coordination and collaboration, making it harder to achieve expected enforcement outcomes. Therefore, the Indonesian maritime security legal framework needs simplification and reform.

There are 10 Indonesian ministries and their subordinate agencies/departments involved in maritime law enforcement [25]. The SATGAS 115 special task force operates under the KKP, is directly accountable to the President, and participates in maritime law enforcement. SATGAS 115 aims to strengthen administrative and legal sanctions against IUU fishing in Indonesian waters. It pioneered the former KKP Minister Susi Pudjiastuti’s ‘sinking vessel’ policy. From its establishment in 2015 to 2016, SATGAS 115 revoked 291 and suspended 261 fishing licenses while issuing 48 warning letters [26].

**Fig 4:**
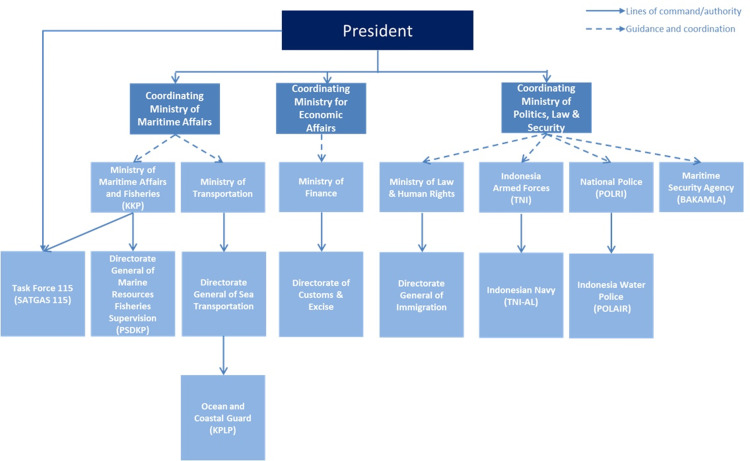
Subordinate relationship of Indonesian enforcement agencies. (Source: Lyle J. Morris and Giacomo Persi Paoli, A Preliminary Assessment of Indonesia’s Maritime Security Threats and Capabilities, Fig 3.3).

The subordinate relationship of the enforcement agencies is illustrated in the following diagram (Fig 4):

Due to the lack of an effective coordination mechanism among Indonesian maritime law enforcement agencies, there are overlaps in functions between different agencies. Taking the relationship between BAKAMLA and Ocean and Coastal Guard (KPLP) as an example—the Shipping Act (Articles 276–278) mandates the formation of KPLP, while the Maritime Affairs Act (Articles 59–63) authorizes the establishment of BAKAMLA, endowing it with nearly identical functions to KPLP [27]. Undoubtedly, the Indonesian government hopes to change the current scenario of multi-agency enforcement by establishing BAKAMLA, as it possesses coordination capabilities and enforcement powers. Some commentators even suggest that “the existence of BAKAMLA will shift the Indonesian maritime enforcement paradigm from multi-agency multi-task to single-agency multi-task.” However, since the Indonesian government has not dismissed other agencies with similar tasks, the establishment of BAKAMLA, to some extent, may have further complicated the overlap of functions among maritime law enforcement agencies.

The above analysis shows that the Indonesian maritime law enforcement legal framework and institutional arrangement are overly complex and riddled with conflicts. Regarding law enforcement agencies, despite the establishment of BAKAMLA and the Indonesian government’s strong desire to make it the leading agency for maritime law enforcement, the lack of effective coordination mechanisms has led to unclear delineation of functions among various agencies, thereby reducing enforcement efficiency. Moreover, this desire faces strong resistance from other maritime law enforcement agencies, as they are concerned that relinquishing enforcement authority could lead to budget cuts and asset transfers. If Indonesia can address this issue and make BAKAMLA the sole maritime enforcement agency, the efficiency and effectiveness of maritime law enforcement would significantly improve.

In contrast, China’s fishery law enforcement framework is primarily based on the Fishery Law, supplemented by other administrative regulations and departmental rules. Regulations such as the Detailed Rules for the Implementation of the Fishery Law of the People’s Republic of China, Regulations on the Management of Distant Water Fisheries, and Regulations on the Management of Fishing Licenses guide the implementation of the Fishery Law. Provisions related to illegal fishing and fishery disputes are scattered throughout the legal framework with no specific legislation or regulations targeting the theme.

In 2013, China restructured the National Oceanic Administration and established the China Coast Guard, unifying various maritime law enforcement agencies and ending the situation of multi-department law enforcement. Prior to this, maritime law enforcement agencies were referred to as the “Five Dragons” [28]. In 2018, the China Coast Guard was further reorganized into the Maritime Police Force under the Chinese People’s Armed Police Force [29]. These changes indicate that, in terms of command and management structure, the China Coast Guard has transformed into a militarily centralized organization commanded by the Central Committee of the Communist Party and the Central Military Commission of China.

The Standing Committee of the National People’s Congress passed the China Coast Guard Law on January 22, 2021, which came into effect on February 1 of the same year. Article 2 of the Coast Guard Law stipulates that the China Coast Guard, as the maritime law enforcement agency, shall uniformly perform the duties of maritime rights protection and law enforcement at sea. Article 83 states that the Coast Guard shall perform defense tasks such as combat operations by relevant laws, military regulations, and orders of the Central Military Commission. It is evident that the China Coast Guard has dual functions: firstly, as a maritime military force engaged in defense and other military tasks in China’s territorial waters (military activities); secondly, to uniformly carry out maritime law enforcement tasks (law enforcement activities), integrating defense and law enforcement missions into one body [30]. However, maritime law enforcement remains the primary responsibility of the China Coast Guard [31]. In the context of the SCS, China has established the SCS Branch of the China Coast Guard, which is primarily responsible for maritime rights protection and law enforcement in the SCS. It supervises, inspects, prevents, and punishes illegal activities in the sovereign waters [32]. Thus far, the China Coast Guard serves as a unified Chinese maritime law enforcement agency. In this regard, China’s current situation surpasses that of Indonesia. However, there may still be a need for coordination among agencies during specific operations since combating illegal fishing or resolving fishery disputes involves different levels of agencies.

#### Policies and measures.

Indonesia’s first policy against IUU fishing was the fishing ban imposed on foreign vessels from October 2014 to April 2015 (later extended to October 2015). During the same year, the KKP prohibited transshipment at sea, even though it could save fuel costs (which typically account for 28% to 60% of total operating costs). In 2015, the KKP issued a regulation prohibiting using unsustainable fishing gear that could harm marine ecosystems. Additionally, the KKP established the “SATGAS 115” Special Task Force Against IUU Fishing in the same year, further intensifying efforts to combat IUU fishing. This task force was highly active in combating IUU fishing, including apprehending foreign fishing vessels near the Natuna Islands. In 2015, SATGAS 115 investigated 1132 “former” foreign fishing vessels [33], finding that all of these vessels were engaged in different degrees of illegal activities [34]. Subsequently, Indonesia blacklisted and deregistered all of them. According to KKP, between November 2014 and May 2019, a total of 516 foreign vessels engaged in IUU fishing were destroyed, including 302 Vietnamese vessels, 91 Philippine vessels, 50 Thai vessels, 41 Malaysian vessels, 27 Indonesian vessels, 2 Papua New Guinea’s vessels, 1 Chinese vessel, 1 Belizean vessel, and one stateless vessel [35].

Meanwhile, Indonesia’s central and local governments have continuously strengthened their fishery law enforcement capabilities. For instance, the fishery department of Bali has approved a Regional Action Plan to promote responsible fishing and combat IUU fishing. This action plan has received technical advice and assistance from 11 countries, including Australia, Brunei, Cambodia, Malaysia, Singapore, and Timor-Leste, as well as four regional fishery management organizations. Furthermore, to enhance enforcement effectiveness, the Indonesian government has taken measures to combat fishery crimes as transnational organized crimes following the United Nations Convention against Transnational Organized Crime (UNTOC). All policies and measures adopted by Indonesia comply with its national strategy of being a ‘global maritime fulcrum’ and support its maritime vision of ‘Sovereignty, Sustainability and Prosperity’ [36].

**Fig 5:**
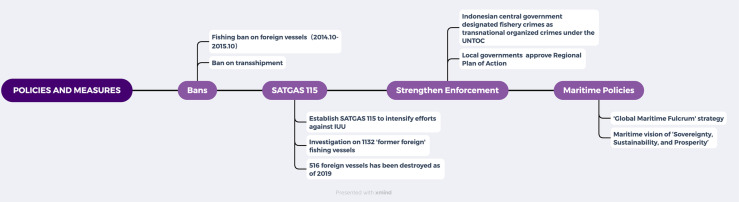
Summary of Indonesian fishery law enforcement policies and measures.

Here is a summary table of the law enforcement measures taken by Indonesia (Fig 5):

To sum up, the Indonesian law enforcement measures against IUU fishing have achieved positive results. Between 2014 and 2017, the growth rate of the fishery GDP was higher than that of the national and agricultural sectors. By the third quarter of 2017, fishery GDP in Indonesia reached 1,695,131 billion Indonesian Rupiah, approximately 11.54 billion U.S. dollars. From 2012 to 2017, the consumption of fish caught kept increasing steadily, with a 37.2% rise in 2017 compared to 2012. It is estimated that if Indonesia continues to implement its current fishery policies and measures, by 2050, the biomass of fishery will increase by 224%, the catch volume will double, and fishery profits will increase by 3.7 billion U.S. dollars [26]. It is evident that Indonesia’s current policies and law enforcement measures against IUU fishing can effectively promote the sustainability of fishery resources.

Measures taken by China are as follows:

First, China has established a Monitoring, Control, and Surveillance (MCS) system. This includes a fishing license system and a fishing vessel registration system for SCS [37], as well as an inspection regime that grants fishery enforcement officers the authority to inspect various documents, fishing gear, catch, and fishing methods of fishing vessels [38]; it requires all distant-water fishing vessels to install vessel monitoring equipment compatible with the Vessel Monitoring System (VMS) [39] and requests the implementation of an observer program [40].

Secondly, China imposes penalties on illegal fishing, including economic sanctions and confiscation of catch and fishing gear. According to Chinese Fishery Law, foreign individuals and fishing vessels engaged in illegal fishing within Chinese jurisdictional waters are ordered by the fishery authorities or fishery supervision and management institutions to leave or would be expelled, and may also face fines and confiscation of catch, fishing gear, and vessels depending on circumstances [41]. Chinese fishermen or fishing vessels engaged in illegal fishing within Chinese jurisdictional waters are also subject to penalties depending on the circumstances. Any fishing vessel that moves, dismantles, closes, or damages the VMS monitoring equipment will have its policy subsidies deducted for that year [42]. A blacklist system is also in place. In 2017, the Chinese Ministry of Agriculture issued a blacklist of distant-water fishing personnel for the first time, and Article 34 of the newly revised Regulations on Distant-Water Fishery Management in 2020 explicitly stipulates the blacklist system.

In July 2019, the Chinese Ministry of Agriculture and Rural Affairs released Suggestions on Further Combating Illegal Fishing Activities, which advocated accelerating the revision of Fishery Law to include illegal fishing within the scope of legal regulation; actively joining and implementing the Port State Measures Agreement; and establishing a specialized team for joint maritime operations against illegal fishing [43]. In fact, to promote the implementation of port state measures, the Ministry of Agriculture and Rural Affairs collaborated with multiple departments, such as the Ministry of Public Security, the Ministry of Foreign Affairs, and the Ministry of Transport. They notified domestic ports of 247 IUU fishing vessels identified by seven regional fishery management organizations China has joined, placing them under surveillance to prevent them from entering Chinese ports [44]. China’s ongoing and active efforts in forming specialized teams and implementing port state measures show its strong will to combat illegal fishing. However, law enforcement against illegal fishing in SCS by China does not match the frequency, intensity, or methods of illegal fishing by surrounding countries. The Chinese law enforcement agencies are highly restrained with illegal fishing vessels and fishermen found within their jurisdiction, rarely detaining fishermen or confiscating fishing vessels, with almost no legal accountability, thereby failing to fundamentally reverse the rights violations and the encroachment of Chinese fishery resources in SCS. In this regard, China could use the Indonesian approach, formulate more precise policies against illegal fishing, and take more effective measures within a reasonable scope.

#### International cooperation.

***International agreements and regional cooperation:*** Indonesia is a party to the United Nations Convention on the Law of the Sea (UNCLOS) and the Port State Measures Agreement (PSMA). UNCLOS outlines the obligation of states to cooperate in the conservation of marine biological resources, while PSMA is the first binding international agreement specifically targeting IUU fishing. As a signatory to both international agreements, Indonesia is obligated to combat IUU fishing.

Additionally, Indonesia has engaged in regional cooperation, such as the 2007 ASEAN Regional Plan of Action to Promote Responsible Fishing including Combating IUU Fishing. This plan set forth specific measures for ASEAN member states to enhance regional cooperation, capacity building, and information sharing in the fight against IUU fishing. Furthermore, Indonesia is a member of three regional fishery management organizations: the Western and Central Pacific Fisheries Commission (WCPFC), the Indian Ocean Tuna Commission (IOTC), and the Commission for the Conservation of Southern Bluefin Tuna. These organizations have established measures for sustainable fishery management and combating IUU fishing.

China is also a member of UNCLOS but has yet to join the PSMA. Nonetheless, China has partially fulfilled its obligations under the PSMA. For instance, China includes the list of IUU fishing vessels published by various regional fishery management organizations in its port surveillance. Chinese fishing vessels that enter foreign ports are encouraged to cooperate with relevant countries on port inspections. China is also advancing port reforms, exploring the establishment of a traceability system for fishery products, and prohibiting the landing of illegal catches. China has prioritized accession to PSMA in its national agenda, demonstrating its proactive stance against IUU fishing. China joins more regional fishery management organizations than Indonesia. Besides the three regional fishery management organizations Indonesia has joined, China has also joined the International Commission for the Conservation of Atlantic Tunas, the Northeast Atlantic Fisheries Commission, the Northwest Atlantic Fisheries Organization, and others, totaling 11 regional fishery management organizations. China has also met the requirements for combating IUU fishing set by these fishery organizations.

***Bilateral law enforcement cooperation:*** Indonesia has engaged in bilateral law enforcement cooperation against IUU fishing with countries such as Papua New Guinea, East Timor, Palau, New Zealand, Australia, and Norway. Take Indonesia’s cooperation with Australia as an example [45]. In February 2017, Indonesia and Australia signed the Joint Declaration on Maritime Cooperation and agreed on the Maritime Cooperation Plan of Action under this declaration. Under the plan, the Australian Navy, the Indonesian Navy, the Australian Border Force, the Indonesian KKP, and BAKAMLA collaborated on multiple joint patrols to combat IUU fishing in their shared borders [46]. For instance, in March 2021, Indonesia and Australia conducted a joint patrol named Gannet 5 under the annual Fisheries Monitoring Forum framework. From the planning phase, both sides held regular online meetings and shared necessary data to ensure the smooth execution of the joint patrol. The mentioned joint patrol was the fifth collaboration in the border area between Indonesia and Australia, and it was to fulfill the requirement of the Maritime Cooperation Plan of Action.

In October 2022, at the 22nd Indonesia-Australia Fisheries Surveillance Forum Annual Meeting held in Darwin, Australia, both states signed the latest cooperation document. The cooperation aims to prevent the threats of IUU fishing to marine and fishery resources, explicitly targeting the boundaries between the Timor Sea and the Arafura Sea [47]. To implement this cooperation, Indonesia and Australia established working groups on key issues related to combating IUU fishing, including public awareness, maritime surveillance and enforcement, and alternative employment opportunities. The maritime surveillance and enforcement group undertook the most critical tasks under the agreement. Its purpose was to ensure the free flow of patrol information in the Arafura and Timor Seas between BAKAMLA and the Australian Maritime Border Command, especially regarding the sharing of intercepted information, to avoid unnecessary vessel collisions.

Indonesia and Australia, considering national security and maritime policy, regard each other as essential partners in maritime cooperation. The successful model of the 1989 Timor Gap Treaty set the tone for subsequent maritime cooperation between the two nations. The joint patrols carried out under the Maritime Cooperation Plan of Action have been effective in combating IUU fishing. The new agreement on IUU fishing in 2022 represents a significant development in their collaboration, creating a favorable political environment for fostering mutual trust and preventing tensions at maritime boundaries.

**Fig 6:**
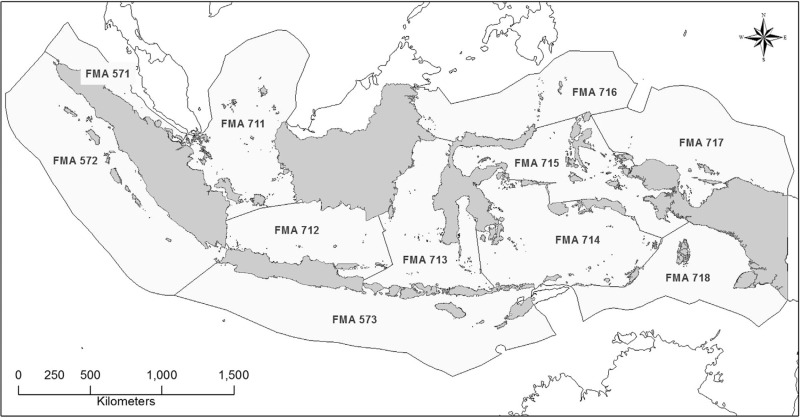
Boundaries of fisheries management area in Indonesia. (Source: Robert S. Pomeroy et al., Review of national laws and regulation in Indonesia in relation to an ecosystem approach to fisheries management, Marine Policy, January 2019, 91(2018), Fig 2).

Take Indonesia and East Timor as another example [48]. On July 20, 2017, Indonesia and East Timor launched the “Cross-Border Cooperation for Promoting Sustainable Marine Management in Indonesia,” led by the FAO and jointly funded by the Global Environment Facility (GEF), the Government of Indonesia, and the Government of East Timor. The project, which ended on December 31, 2023, was carried out by Indonesian KKP and East Timor’s Ministry of Agriculture and Fisheries. The project had three main objectives: Firstly, identify and address marine environmental threats, including unsustainable fishing practices. Secondly, enhance regional and sub-regional cooperation capacity in marine resource management. Lastly, coordination between regional information networks, project effectiveness monitoring, and information dissemination. The third objective included strengthening cooperation in monitoring and reporting IUU fishing. According to the implementation report jointly released by the FAO and GEF in 2022, the project improved the monitoring and reporting of IUU fishing within the Indonesian Sea Large Marine Ecosystem and promoted cooperation against IUU fishing with adjacent large marine ecosystems and countries. For instance, the two states held national conferences on transboundary fishery issues to develop fishery resource monitoring strategies based on Indonesian fishing areas (712, 713, 714, and 573) (Fig 6) to support the measurable fishery requirements of both countries. They also planned to cooperate in combating IUU fishing in Indonesia, with training on data usage to monitor threats to fishery resources. Also, efforts are made to integrate East Timor into the Indonesian data system. For Indonesia, the project also enhanced coordination among its domestic institutions aimed at combating IUU fishing [49].

China has initiated bilateral fishery law enforcement cooperation with countries like Indonesia, South Korea, Russia, and Vietnam. These collaborations often do not explicitly prioritize illegal fishing as their primary objective. Instead, they focus on enhancing the capabilities of marine governance and maritime cooperation, which indirectly positively impacts combating illegal fishing and dealing with fishery disputes

In 2013, China and Indonesia elevated their relationship to a comprehensive strategic partnership. This framework agreement covered many aspects of bilateral relations and laid the groundwork for deeper cooperation, including in the fishery sector. The same year, both parties signed a Memorandum of Understanding (MoU) on fishery cooperation. The MoU emphasized sharing fishery data, particularly data related to imports and exports of fishery products, as well as vessel registration and de-registration information. Additionally, the two countries proposed the establishment of a joint commission on maritime affairs and held multiple discussions on this initiative.

In the context of fishery cooperation in the Beibu Gulf between China and Vietnam, both sides have conducted joint patrols to combat illegal fishing and promote the sustainable use of marine resources [50]. However, the cooperation covers a relatively small area. Additionally, due to historical overlaps in traditional fishing grounds between China and Vietnam, fishermen have struggled to adapt to new operational regulations after the signing of the agreement. Consequently, fishing vessels from both countries continue to conduct cross-border fishing occasionally, significantly reducing the effectiveness of the conservation efforts under the agreement.

Compared to Indonesia, Chinese bilateral fishery law enforcement cooperation remains relatively superficial. They often appear in forms like exchanges and training of fishery technology, as well as joint surveys of fishery resources. There has been limited substantive cooperation in fishery law enforcement. To adequately address illegal fishing and fishery disputes in SCS, China needs to strengthen fishery law enforcement cooperation with other SCS coastal states. However, achieving this goal is complicated by unresolved maritime disputes among SCS coastal states and interference from external actors. Therefore, multiple factors must be considered before China can develop a feasible strategy.

#### Law enforcement in disputed waters.

Taking the Chinese SCS moratorium and Indonesian law enforcement actions around the Natuna Islands as examples to compare the differences and impacts of law enforcement measures in disputed waters of these two countries may provide insights for China.

Indonesia’s law enforcement actions around the Natuna Islands have been notably strict. For instance, in 2020, the Indonesian government discovered several Chinese fishing vessels operating in the waters near the Natuna Islands. In response, the Indonesian Navy dispatched multiple warships and fighter jets to the area to enforce its laws against the Chinese vessels. Indonesian President Joko Widodo personally visited the Natuna Islands, where he announced strengthening naval and coast guard deployments in the region, underscoring Indonesia’s sovereign claims and determination of law enforcement over the Natuna Islands. This incident led to diplomatic tensions between China and Indonesia, but Indonesia maintained the legitimacy of enforcing laws within its EEZ. Since 2014, the Indonesian government has adopted strict measures, including sinking measures, to deter illegal fishing activities, particularly around the Natuna Islands. In 2019, Indonesia sank several illegal fishing boats from Vietnam and other countries in the waters around the Natuna Islands. In response, the Vietnamese government lodged a formal diplomatic protest. It emphasized that Vietnamese fishermen were operating within Vietnam’s traditional fishing grounds and called for the two countries to resolve their differences through negotiations rather than unilateral law enforcement actions.

Chinese law enforcement measures during the SCS fishing moratorium include increasing the frequency of maritime patrols, especially in key fishing grounds and areas prone to IUU fishing. Inspections are conducted at major ports and docks to ensure that docked vessels comply with the moratorium regulations. Fishing vessels are required to activate their vessel tracking systems when going to sea, allowing enforcement agencies to monitor their positions and ensure they are not engaging in fishing activities during the moratorium. For vessels found violating the regulations, Chinese enforcement agencies may impose economic penalties, confiscate fishing gear and vessels, and, for repeat offenders, revoke their fishing licenses. In cases of serious violations, responsible individuals may be handed over to judicial authorities for criminal prosecution. In May 2024, as the SCS entered the summer fishing moratorium, the China Coast Guard’s SCS Bureau launched a special enforcement campaign. By May 7, they had deployed 617 enforcement vessels, inspected 82 fishing vessels, prosecuted 11 fishing vessels, handed over 3 of these vessels for further action, expelled 76 foreign fishing vessels, and initiated one criminal case [51]. Under these stringent enforcement measures, maritime safety and the fishing moratorium were effectively maintained during the moratorium period.

Chinese enforcement measures during the SCS fishing moratorium have effectively reduced fishing pressure, giving fish populations ample time to recover, which has led to increased catch yields for fishermen during the non-moratorium periods. The strict enforcement actions and administrative penalties have maintained a strong deterrent against IUU fishing, significantly reducing such activities during the moratorium. China has widely applied modern technologies in law enforcement during the moratorium, such as drone patrols, remote sensing, vessel tracking systems, and electronic monitoring platforms. These technologies have made law enforcement more efficient and precise, greatly expanding the range and capabilities of law enforcement and thereby modernizing maritime law enforcement methods. Furthermore, China has actively engaged in joint enforcement and information-sharing with neighboring countries. In August 2021, China conducted joint patrols with the Vietnamese Coast Guard in the northern part of the SCS, repatriating several foreign fishing vessels suspected of IUU fishing. Such cooperation contributes to the sustainable use of regional fishery resources and strengthens trust and collaboration between China and neighboring countries in the SCS. Regarding concerns about the overlap of the moratorium area with the EEZs claimed by other SCS coastal states, China asserts that its fishing moratorium is in strict compliance with the UNCLOS and aligns with international norms. The moratorium is not intended to alter the existing international order or unilaterally impose influence on other countries; rather, it is intended to collaborate with coastal states in the SCS to protect the region’s ecological environment and fishery resources jointly. China is open to sincere dialogue and exchanges with SCS coastal states regarding implementing the moratorium and is willing to work through cooperative mechanisms to address any disputes or misunderstandings that may arise from it.

Upon comparison, Chinese fishery law enforcement in disputed waters is more restrained, with a greater emphasis on regional stability. While the sovereignty of the Natuna Islands is not disputed, the surrounding waters are connected to the traditional fishing grounds of other SCS coastal states. Therefore, law enforcement measures in such areas should remain within a certain degree, avoiding excessive use of force or extreme measures to prevent negative impacts on regional peace and stability. China could achieve a consensus with other SCS coastal states on fishery resource management through increased diplomatic dialogues and regional cooperation, which will ensure that the rights of all parties are respected and protected while avoiding tensions that could arise from law enforcement measures.

### Chinese paths

#### Clear legislation, policies, and institutional arrangement.

Indonesia has complex maritime security legislation, with conflicts between laws, presenting a state of “over-regulation.” Moreover, there needs to be a clearer distribution of authority among various enforcement agencies. As stated above, while Indonesia has established BAKAMLA to handle most maritime security and safety matters, it has yet to explicitly state whether BAKAMLA has law enforcement power. Other maritime enforcement agencies also resist ceding their powers and equipment to BAKAMLA to prevent their authority from being weakened. Although the Indonesian government intends to elevate BAKAMLA to the Indonesian National Coast Guard, the intention is not reflected in legislation. Therefore, a significant function overlap exists between BAKAMLA and the existing KPLP, leading to resource wastage and reducing maritime law enforcement efficiency.

For China, the chaotic situation before 2013 significantly improved with the promulgation of the Coast Guard Law [52], which designated the Coast Guard as the only agency responsible for maritime law enforcement, providing a unified top-level framework for maritime law enforcement. According to Article 2 of the Coast Guard Law, the Coast Guard agencies include the China Coast Guard, its maritime branch bureaus and directly affiliated bureaus, provincial coast guard bureaus, municipal coast guard bureaus, and coast guard stations. China should differentiate sole responsibility tasks from cooperative tasks to avoid unclear authority distribution among enforcement agencies. Complex tasks such as combating illegal fishing and resolving fishery disputes can be designated as cooperative tasks. Simpler tasks can be handled independently by each agency. In this way, wastage of law enforcement resources can be avoided, and law enforcement efficiency will be enhanced.

#### Take more effective measures.

Currently, Chinese law enforcement efforts to combat illegal fishing do not match the frequency, intensity, or methods of illegal fishing by neighboring countries and, therefore, cannot effectively curb illegal fishing in its EEZ. China may consider adopting more effective enforcement measures. On April 4, 2020, the Vietnamese fishing vessel QNG90617TS illegally entered the waters of China’s Xisha Islands for illegal fishing. The Chinese Coast Guard vessel 4301 issued a warning to drive it away by relevant laws. However, the Vietnamese fishing vessel refused to leave and repeatedly made dangerous maneuvers, eventually colliding with and sinking after hitting the Chinese Coast Guard vessel 4301. The Chinese Coast Guard rescued all eight crew members on the Vietnamese vessel [53]. There have also been a lot of fishery disputes between China and Philippines [54]—on August 19, 2024, two Philippine Coast Guard vessels, 4410 and 4411, illegally entered waters near Xianbin Reef in the Spratly Islands without permission from the Chinese government. They ignored China’s repeated stern warnings and were finally controlled by the China Coast Guard by laws and regulations [55]. In cases where repeated warnings are ignored, China could adopt strict enforcement measures, including sinking vessels. However, the prerequisites for adopting such measures should be clearly defined to ensure that they do not violate the necessity requirement of Article 73(1) of UNCLOS.

Article 73(1) of UNCLOS clarifies coastal states’ law enforcement rights within their EEZ, stating that coastal states may take necessary measures, including boarding, inspection, arrest, and judicial proceedings, to exercise their sovereign rights within their EEZs and ensure compliance with their laws in accordance with UNCLOS. Although Article 73(1) does not explicitly mention sinking vessels and a uniform standard for such measures has yet to be established, countries including Malaysia, France, Australia, and the United States have taken measures to sink vessels engaged in illegal fishing within their EEZs. In the M/V Virginia G case, ITLOS clarified what constituted “necessary enforcement measures.” Although the case was about vessel confiscation, it provided guidance on the necessity of sinking measures. The tribunal held that the interpretation of Article 73(1) should be based on “punitive measures by coastal states against illegal fishing.” The tribunal adopted a broad interpretation, without limiting “necessary” to “indispensable,” “absolutely necessary,” or “unavoidable,” but interpreted “necessary” as a middle ground between “unavoidable” and “helpful.” From the text itself, the word “including” in Article 73(1) indicates that “boarding, inspection, arrest, and judicial proceedings” are part of the measures that can be taken; they are not exhaustive. Thus, UNCLOS does not prohibit member states from taking measures such as sinking vessels to combat IUU fishing. Judge Paik stated in his separate opinion that if a choice could be made from several appropriate measures, the least harmful to others’ rights and equally effective measures should be chosen. The enforcement measures taken by coastal states against illegal fishing can only be overturned in cases of clear unreasonableness or extreme malevolence. However, due regard should be given to the rights of other states when taking such measures.

Many Indonesian scholars argue that the Indonesian measure of sinking vessels is fully compliant with Indonesian domestic law as well as international maritime law, as represented by UNCLOS. However, issues with these measures primarily lie in their appropriateness rather than their legality. Under the leadership of Susilo, the predecessor of Joko Widodo, Indonesia had begun to restore its reputation as a leading ASEAN nation. Although the Joko administration asserted that the sinking measures were a last resort after ineffective communication with neighboring countries, such strict measures had been criticized by ASEAN members. They believed that these actions undermined the solidarity of ASEAN [56].

As for China, measures such as sinking vessels should not be easily taken in order to avoid exacerbating tensions in SCS. When it is necessary to take such measures, a “necessity test” should be established. Such a standard should strike a balance between China’s sovereignty and economic interests and those of other SCS coastal states. As mentioned, China has implemented a fishing moratorium to protect fishery resources and the environment in SCS. However, the areas covered by this moratorium overlap with the EEZs claimed by other SCS coastal states, creating some ‘disputed waters’, so the policy has met resistance from some of these countries. For example, Vietnam issues statements opposing China’s annual announcement of the fishing moratorium, arguing that it infringes on ‘Vietnam’s sovereignty over the Spratly Islands and surrounding waters.’ In 2019, a Vietnamese fishing vessel was seized by China during the moratorium period, prompting protests from Vietnam, which claimed that China’s actions were an attempt to unilaterally extend its maritime enforcement powers. China’s fishing moratorium is based on the sovereign rights granted to coastal states within their EEZs under the UNCLOS, aligning with the practices of other coastal states, and is thus legitimate. The moratorium is crucial for the sustainable development of fishery resources in the SCS, benefiting not only China but also other SCS coastal states. How to appropriately conduct enforcement measures without invoking tensions is a crucial problem that China should reflect on when conducting law enforcement operations within disputed waters.

#### Reinforce international cooperation.

Reviewing the evolution of the UNCLOS, contracting states are obliged to cooperate on a wide range of issues. Of particular note, it sets cooperation obligations for semi-enclosed seas coastal states [57]. To fulfill the requirements of the international maritime law, SCS coastal states should actively engage in international cooperation.

China is not only a signatory to UNCLOS but is also actively pursuing accession to the Agreement on Port State Measures (PSMA). Under the frameworks of RFMOs that it has joined or signed bilateral agreements, China has also cooperated with other countries to combat illegal fishing. In this sense, China is actively fulfilling its international obligations to conserve marine resources. However, as previously mentioned, the current Chinese international cooperation in maritime law enforcement remains relatively superficial. In line with the longstanding Chinese principle of “shelving disputes and pursuing joint development” in disputed waters in SCS, China could consider “shelving disputes and jointly conserving fishery resources.” In disputed waters with frequent fishery disputes, relevant coastal states could establish provisional arrangements such as common fishing areas following Article 74(3) of UNCLOS.

Taking the Natuna Islands as an example, according to statistics, only 11.31% of the fishery resources near the Natuna Islands are being exploited. The rich fishery resources surrounding the islands could become a focal point of contention among coastal states with overlapping claims, increasing the risk of conflicts [58]. Analysis of fishing intensity shows a significant increase in fishing intensity around the Natuna Islands, gradually intensifying from south to north. In other words, the closer to the overlapping part of the Indonesian EEZ and the Nine-Dash Line, the more pronounced the increase in fishing intensity (see Fig 1), which to some extent indicates the possibility of fishery conflicts between China and Indonesia in the vicinity of the Natuna Islands. In recent years, the Natuna Islands have been rapidly militarized, and there have been several maritime standoffs between China and Indonesia [59,60]. In November 2022, then Commander-in-Chief of the Indonesian National Armed Forces, General Andika Perkasa, stated in an interview that Indonesia would conduct joint military exercises with Malaysia and Brunei around the Natuna Islands [61] to ensure Indonesian sovereignty and economic interests. It is evident that the Chinese Nine-Dash Line claim may provoke Indonesia’s sensitive sovereignty concerns. China should seek to reconcile this contradiction to avoid direct conflicts with Indonesia over fishery resources around the Natuna Islands. China can draw lessons from the agreement with Vietnam on the Beibu Gulf joint fishery zone and try to establish joint fishery zones, transitional waters, and buffer zones for fishing vessels with Indonesia in disputed areas around the Natuna Islands. The scope of such areas can be determined upon where fishing intensity has significantly increased around the Natuna Islands, combined with the overlapping parts of the Indonesian EEZ and Chinese Nine-Dash Line.

It is worth noting that bilateral or regional cooperation must be established upon mutual political trust between the regions or countries involved; otherwise, cooperation may be hindered by political factors. Take the bilateral cooperation between Indonesia and Australia as an example. After the Australian surveillance incident in 2013, Indonesia suspended all military and intelligence cooperation with Australia. It was not until 2014, when they signed the “Joint Understanding on a Code of Conduct,” that their bilateral relations were restored. In 2016, Indonesia suspended joint military training after materials allegedly insulting Indonesia’s five founding principles - Pancasila - were found at an Australian training base. Full military cooperation between the two countries was resumed in February 2017 after Australia issued an apology and made commitments [62]. One thing that may hamper the trust between China and other SCS coastal states is that the United States identified China as a “nation engaged in or supporting IUU fishing”. This is a groundless opinion because the subjects engaged in IUU fishing are fishing vessels, and the illegal activities of individual fishing vessels should not be attributed to states. China should make it clear to other SCS coastal states to dispel unnecessary doubts.

#### Enhance participation of local fishing communities.

One of the causes of IUU fishing is weak governance. For instance, ineffective law enforcement by Indonesian local governments fails to extend central government policies to their respective jurisdictions, resulting in frequent occurrences of IUU fishing. Fishermen in IUU fishing-affected areas often face more severe economic difficulties. In desperate situations, some fishermen may risk venturing further to more prosperous fishing grounds despite the risks associated with small fishing vessels sailing far from shore. Others may turn to IUU fishing or other maritime crimes such as piracy, smuggling and maritime terrorism. Consequently, while being victims of IUU fishing, some fishing communities may also become perpetrators [63], thus perpetuating a vicious cycle that exacerbates IUU fishing in various regions. Research conducted on Indonesian local fishing communities indicated that the majority feel they do not receive sufficient support from local governments and cannot rely on them to address the livelihood and safety threats posed by IUU fishing. At the same time, the central government seems too distant to address their concerns. In cases where local governments fail to act, some fishing communities have no choice but to take law enforcement into their own hands and destroy illegal fishing vessels themselves. The Presidential Decree No. 16 of 2017 concerning Indonesian Ocean Policy did not identify local governments and local fishing communities as primary beneficiaries, nor did it clearly define the roles of local governments and local fishing communities in combating IUU fishing. Consequently, in Indonesian national-level design and law enforcement practices, the potential role of local fishing communities has been overlooked, resulting in a disconnect between law enforcement measures and the realities on the ground. KKP has established a social institution under the Directorate-General of Surveillance and Control of Marine and Fishery Resources (PSDKP), known as the Monitoring Community Group, to address this issue. This group is responsible for engaging surrounding communities, particularly local fishing communities, to assist the Indonesian government in combating IUU fishing. The primary responsibility of this institution is to incorporate the capabilities and insights of local communities to review and protect fishery resources, as local communities are often people who are most familiar with the conditions of the environment and resources [64].

Although the role of local fishing communities in combating IUU fishing is internationally recognized, in practice, these communities often do not receive the attention they deserve, even if they are the most knowledgeable people about the methods to combat IUU fishing. Therefore, integrating local fishing communities into the construction of Chinese fishery law enforcement system, listening to their opinions and suggestions, and establishing cooperation channels between the government and local fishing communities should be an essential direction for China.

## Conclusion

The overfishing crisis in the SCS is characterized by alarming statistics highlighting resource depletion severity. According to various studies, fish stocks in the SCS have declined by over 70% in the past few decades. The region, which supports around 12% of the global fish catch, has seen its annual fish catch drop from approximately 10 million tons in the 1990s to less than 5 million tons in recent years. IUU fishing is rampant in SCS, accounting for up to 30% of the total catch in the area. Developing countries along SCS, lacking sufficient capacity to address IUU fishing, are more vulnerable to this issue. Moreover, fishery disputes in disputed waters are making the situation worse. Therefore, there is a considerable necessity for SCS coastal states to improve law enforcement systems to properly handle IUU fishing and fishery disputes so that overfishing in SCS would not exacerbate.

Indonesia, the largest archipelagic country in the world, endeavored to build its enforcement system against IUU fishing, which has achieved considerable success. The article compares the law enforcement systems of Indonesia and China from four main aspects: legal frameworks and institutional arrangements, policies and measures, international cooperation and law enforcement in disputed waters. The analysis suggests that China’s current legal framework and institutional arrangement are superior to Indonesia’s complex maritime security system but emphasizes the importance of coordination among participating agencies during specific operations. Regarding policies and measures, the Indonesian system has achieved more notable results but lacked attention to regional stability, indicating that China should formulate clearer policies and adopt more effective measures without invoking tensions among SCS coastal states. Regarding international cooperation, both countries have joined several regional fishery management organizations. However, China could pursue deeper law enforcement cooperation based on political mutual trust at the bilateral level. As for law enforcement in disputed waters, China is more restrained in applying strict measures and pays more attention to regional stability.

In light of the comparative analysis, China should focus on improving its fishery law enforcement system in SCS from the following four aspects: Firstly, clear legislation, policies, and institutional arrangements. While the Coast Guard Law and the Coast Guard currently serve as the top-level design and unified maritime law enforcement agency of China, the distribution of authority among the Coast Guard’s subordinate divisions should be clarified to avoid overlaps or gaps in law enforcement. Secondly, effective law enforcement measures complying with Article 73(1) UNCLOS. More effective law enforcement measures should be implemented while considering the necessity requirements of Article 73(1) UNCLOS. It would allow China to safeguard its sovereignty or sovereign interests without exacerbating tensions in SCS. Thirdly, reinforce international cooperation. Actively building political mutual trust with SCS coastal states and considering the approach of “shelving disputes and jointly conserving fishery resources” would be crucial. Lastly, the participation of local fishing communities should be enhanced. Local fishing communities are the most knowledgeable people of IUU fishing methods and potential solutions. Their involvement can provide valuable insights and enhance the effectiveness of law enforcement efforts.

Addressing overfishing is vital for all SCS coastal states. By implementing the above schemes, hope China could ultimately establish a comprehensive, efficient, and balanced fishery law enforcement system against overfishing in SCS.

## Supporting information

S1 File Results of figures. (ZIP)
